# Noise and Body Fat: Uncovering New Connections

**DOI:** 10.1289/ehp.124-A57

**Published:** 2016-03-01

**Authors:** Wendee Nicole

**Affiliations:** Wendee Nicole was awarded the inaugural Mongabay Prize for Environmental Reporting in 2013. She writes for *Discover*, *Scientific American*, *National Wildlife*, and other magazines.

Studies on environmental noise and human health have uncovered associations with cardiovascular disease^1^^,^^2^ and diabetes.^3^ New research is delving further into possible metabolic effects of noise—specifically a possible link to weight gain. In this issue of *EHP* investigators report that exposure to traffic noise at home was associated with body composition outcomes such as larger waist circumference and higher body mass index (BMI).^4^

The cross-sectional study used data from the Danish Diet, Cancer, and Health Cohort, assessing 52,456 Danes between the ages of 50 and 64. The study tracked each participant’s residential address history for the previous 5 years. The authors used noise-mapping software to estimate exposures from road traffic, railways, and air traffic for each address based on the most noise-exposed façade of the home. Four measures of body composition were recorded for each participant—BMI, waist circumference, body fat mass index (BFMI), and lean body mass index (LBMI).

**Figure:**
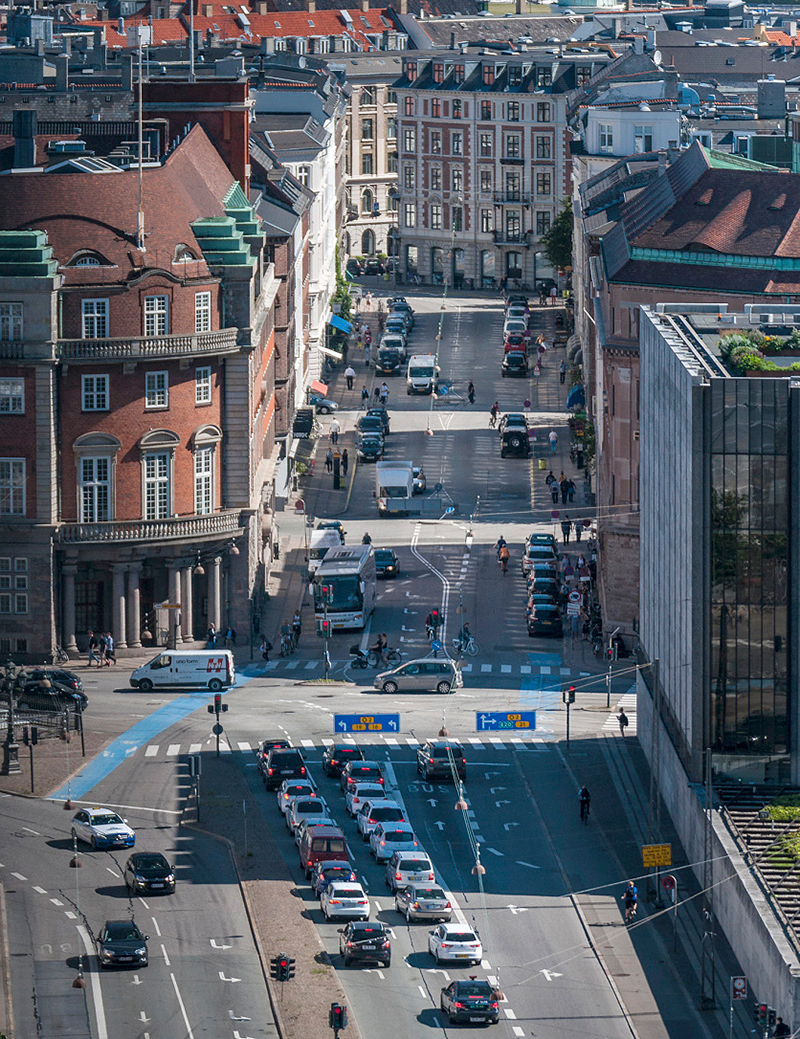
Residential exposure to traffic noise has been associated with measures of weight gain. The body’s response to both stress and lack of sleep may help explain why. © Dorte Fjalland/Getty

After adjusting for potential confounding factors (socioeconomic status, age, sex, and exposure to railway and aircraft noise), the researchers found that all measures of adiposity were significantly associated with road traffic noise. Each 10-dB increase in average road traffic noise exposure over 5 years was associated with an average increase in waist circumference of 0.35 cm and an average increase in BMI of 0.18 points. BFMI and LBMI also showed small but statistically significant increases in association with greater road traffic noise exposure. Co-exposure to railway noise louder than 60 dB appeared to heighten the associations with BMI, waist circumference, and BFMI.^4^

“The linear association we observed was consistent throughout the exposure range,” says lead author Jeppe Christensen, a PhD candidate in epidemiology with the Danish Cancer Society Research Center. This is in line with other studies of similar health effects.

The authors propose that noise may activate the hypothalamus–pituitary–adrenal axis and the sympathetic nervous system—the body’s “fight or flight” response. Evidence for this mode of action from other studies includes increased levels of cortisol associated with exposure to louder road noise.^5^ Noise may also disturb sleep, which is associated with increased food intake,^6^ possibly due to dysregulation of hunger-related hormones, including leptin and ghrelin.^7^^,^^8^ Epidemiological studies have also reported that lack of sleep in children and young adults is associated with a higher percentage of body fat and increased waist circumference.^9^

A major strength of the study was its sheer size, and according to Bente Oftedal, an epidemiologist at the Norwegian Institute of Public Health, the results and conclusions matched the rigor of the performed analyses. “The main weakness is the lack of data on noise-related individual characteristics, such as noise annoyance and noise sensitivity,” she says. “Both characteristics may modify associations between traffic noise and health outcomes, representing vulnerable subpopulations to noise exposure.” Oftedal was not involved with the study.

“This is one of only a handful of studies investigating the association between exposure to noise in the environment and metabolic effects,” says Charlotta Eriksson, a researcher at the Karolinska Institute’s Institute of Environmental Medicine in Stockholm, who led one of the first studies to link aircraft noise with obesity.^10^ “The study by Christensen therefore adds valuable knowledge into this field of research.”

The estimated effects of noise are small, Eriksson adds, but she says this is to be expected because other risk factors, such as heredity and lifestyle factors, are much stronger predictors of obesity for the individual. “Nevertheless,” she says, “since a large proportion of the population is exposed to road traffic noise, the public health impact may be substantial.”
